# Host-microbe interactions in the gut of *Drosophila melanogaster*

**DOI:** 10.3389/fphys.2013.00375

**Published:** 2013-12-17

**Authors:** Takayuki Kuraishi, Aki Hori, Shoichiro Kurata

**Affiliations:** ^1^Department of Molrcular Biopharmacy and Genetics, Graduate School of Pharmaceutical Sciences, Tohoku UniversitySendai, Japan; ^2^PRESTO, Japan Science and Technology AgencyTokyo, Japan

**Keywords:** *Drosophila*, innate immunity, peritrophic matrix, antimicrobial peptide, reactive oxygen species, intestinal stem cell, gut flora

## Abstract

Many insect species subsist on decaying and contaminated matter and are thus exposed to large quantities of microorganisms. To control beneficial commensals and combat infectious pathogens, insects must be armed with efficient systems for microbial recognition, signaling pathways, and effector molecules. The molecular mechanisms regulating these host-microbe interactions in insects have been largely clarified in *Drosophila melanogaster* with its powerful genetic and genomic tools. Here we review recent advances in this field, focusing mainly on the relationships between microbes and epithelial cells in the intestinal tract where the host exposure to the external environment is most frequent.

## Introduction

Microorganisms exist in all parts of the biosphere, which exerts pressure on multicellular organisms to develop mechanisms to effectively respond to microbes. In humans, for example, as much as 2 kg of indigenous bacteria are thought to be contained in the intestine, the respiratory tract, the genitals, and on the skin, an amount that corresponds to 10 times the number of host cells. The gut epithelium, the body part frequently exposed to the external environment, contains the majority of the commensals in the body. Intestinal bacteria benefit the host by gleaning energy from the fermentation of undigested carbohydrates and facilitating the subsequent absorption of short chain fatty acids by the host. They also play a role in synthesizing vitamins and in metabolizing bile acids and sterols (Cummings and MacFarlane, 1997). Recent studies revealed that microbiota also contribute to maintain host homeostasis and immune responses. Deregulated alterations of microbiota induce chronic inflammation in the intestine (Sartor, 2004; Round and Mazmanian, 2009), as well as cancer in the liver (Yoshimoto et al., 2013).

Because the gut epithelium is frequently exposed to harmful pathogens, it must be armed with an efficient and powerful immune system to protect itself. The fruit fly *Drosophila melanogaster* possesses a gut that is structurally and functionally similar to mammalian intestinal tract (Lemaitre and Miguel-Aliaga, 2013), which is constantly in contact with microbial pathogens as flies ingest large quantities of microorganisms through feeding on rotting fruits. *Drosophila* is a powerful model organism for deciphering innate immune responses (Lemaitre and Hoffmann, 2007). Analysis of systemic immune responses of *Drosophila* in response to the direct invasion of pathogens into the body fluid revealed that the Toll pathway is important for fungal infection and led to the discovery of the mammalian Toll-like receptor (TLR) signaling pathway (O'Neill et al., 2013). TLR signaling is crucial to the first line of defense against pathogens as well as the induction of adaptive immunity (Iwasaki and Medzhitov, 2010; Takeuchi and Akira, 2010). Recent studies in *Drosophila* have begun to reveal the mechanisms that regulate gut defense against bacterial infection and provide insights into the mammalian intestinal defense system (Royet, 2011; Buchon et al., 2013a; Ferrandon, 2013). *Drosophila* gut defense responses comprise four steps: (i) physical barriers, such as the peritrophic matrix and epithelial integrity (Hegan et al., 2007; Bonnay et al., 2013), (ii) production of reactive oxygen species (ROS), (iii) secretion of antimicrobial peptides (AMPs) into the hemolymph through the Imd pathway, and (iv) epithelium renewal to maintain homeostasis in response to gut damage. In the first half of this review, we describe these four steps in more detail.

On the other hand, *Drosophila* also possess indigenous gut microbiota that have important roles in the host physiology and pathology (Broderick and Lemaitre, 2012). How flies discriminate benign and beneficial bacteria from pathogenic microbes to maintain harmonious gut flora, however, has remained unclear. In the latter part of this review, we describe the importance of the ROS-producing pathway and negative regulators of the Imd pathway for maintaining commensals, and discuss possible future directions of the gut immunology.

## Peritrophic matrix as a physical barrier of gut epithelium

**Figure 1:**
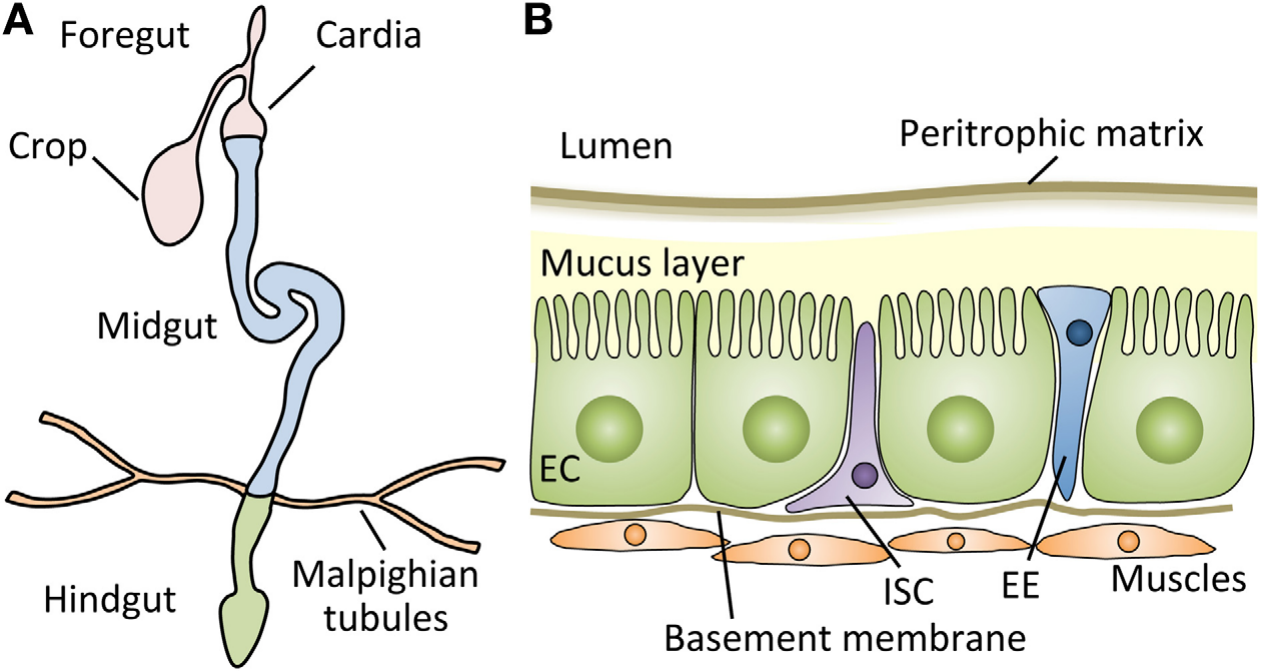
**The structure of the *Drosophila* gut. (A)** Schematic representation of the digestive tract of the *Drosophila* adult. The *Drosophila* gut is a tubular epithelial organ composed of a monolayer of cells surrounded by muscles. The gut is divided into the foregut, the midgut, and the hindgut, based on the developmental origin. The crop stores the food ingested by the flies, and the first half of the cardia belongs to the foregut. The Malpighian tubules, a functional equivalent of the mammalian kidney, connect at the midgut-hindgut junction. The midgut is the main site of digestion, and most studies of *Drosophila* gut immunity have focused on this compartment. **(B)** A cross section of the adult *Drosophila* midgut. The adult midgut contains several types of cells: absorptive enterocytes (ECs), secretory enteroendocrine cells (EEs), and pluripotent intestinal stem cells (ISCs). Muscle cells are present under the basement membrane of epithelial cells. Between the lumen and epithelia, a semipermeable non-cellular structure, the peritrophic matrix, protects the enterocytes from abrasive particles and pathogens. In addition, a mucus layer lies between the peritrophic matrix and ECs along the midgut (Ridley et al., 2012).

The peritrophic matrix is an acellular structure that forms a layer comprising chitin polymers and glycoproteins, such as peritrophins, lining the insect midgut lumen (Figure 1B) (Lehane, 1997; Hegedus et al., 2009). The peritrophic matrix, though structurally different, is considered analogous to the mucus layer of the mammalian digestive tract, and shields the midgut epithelium from abrasive food particles and microbes (Hegedus et al., 2009). Indeed, studies in insects suggest that the peritrophic matrix protects hosts from xenobiotics and toxins, such as dichloro-diphenyl-trichloroethane (DDT) and Bacillus thuringiensis (Bt) toxins (Tellam, 1996; Hayakawa et al., 2004). Interestingly, *Plasmodium* species secrete chitinases that disrupt the peritrophic matrix, which facilitates their passage through the midgut epithelia in mosquitos (Abraham and Jacobs-Lorena, 2004).

Drosocrystallin (Dcy) protein is a recently identified component of the peritrophic matrix in the adult *Drosophila* midgut, and genetic evidence suggests a protective role of the peritrophic matrix against pathogens (Kuraishi et al., 2011). *dcy* encodes a chitin-binding protein and its expression is upregulated upon the ingestion of bacteria. A strong loss-of-function mutation in *dcy* reduces the peritrophic matrix width by half and increases its permeability to larger molecules, suggesting that, despite the high number of structural proteins associated with the peritrophic matrix, Dcy is an essential component of the peritrophic matrix in the *Drosophila* adult. Moreover, *dcy*-deficient flies exhibit increased susceptibility to oral infections by the entomopathogenic bacteria *Pseudomonas entomophila* (Vodovar et al., 2005) and *Serratia marcescens*. *dcy* mutant flies also succumb faster than wild-type flies upon ingestion of a *P. entomophila* extract. This lethality is due in part to Monalysin (Opota et al., 2011; Blemont et al., 2013), a pore-forming toxin produced by *P. entomophila*. Furthermore, the ingestion of bacteria induces a higher level of expression of AMPs in the *dcy* mutant (Kuraishi et al., 2011), implying that the peritrophic matrix modulates immune responses in the gut.

Though the precise mechanism underlying transcriptional control of the *dcy* gene remains to be determined, microarray analysis comparing the transcriptome following ingestion of non-lethal *Erwinia carotovora* with that of lethal *P. entomophila* revealed that *dcy* induction upon the ingestion of the lethal bacteria is approximately four times higher than that of the non-lethal bacteria (Chakrabarti et al., 2012), suggesting that *dcy* expression is regulated by the degree of stress or damage to the gut.

The peritrophic matrix is a semipermeable membrane, and its permeability is partly controlled by protein-protein cross-linking by transglutaminase in *Drosophila* (Shibata et al., 2013). Along this line, in the mosquito *Anopheles gambiae*, the mucus layer, which is cross-linked by dityrosine covalent bonds, and the peritrophic matrix, control gut permeability. Peroxidase/dual oxidase (Duox) forms this dityrosine network to decrease gut permeability to immune elicitors from *Plasmodium*, thereby preventing the activation of immune responses in the gut (Kumar et al., 2010). Curiously, *dcy* is exclusively expressed in the adult stage, though *Drosophila* larvae also have a peritrophic matrix, and no *dcy* homolog has been identified outside the *Drosophilidae* family. The extreme diversity of insect species in terms of their mode of life and way of feeding, leads them to encounter different kinds of microbes and abrasive particles. Distinct compositions of the peritrophic matrix and dynamic modifications of its permeability by protein-protein cross-linking in each insect species or at different life stages might allow them to adjust to their various environments by ensuring a protective role of the peritrophic matrix against invaders.

## Production of reactive oxygen species following oral infection

NADPH oxidase family proteins are membrane-bound enzymes that catalyze the generation of ROS. They are localized in the plasma membrane as well as in the phagosomal membrane. NADPH oxidases in phagosomes kill engulfed bacteria in neutrophils and macrophages (Babior, 1999). Duox is an enzyme belonging to the NADPH oxidase family proteins in *Drosophila*, and is likely to be expressed in the plasma membrane. Duox is an important factor responsible for gut defense (Ha et al., 2005; Bae et al., 2010). In fact, *Duox*-RNAi flies are susceptible to the ingestion of yeast and several bacterial species.

**Figure 2:**
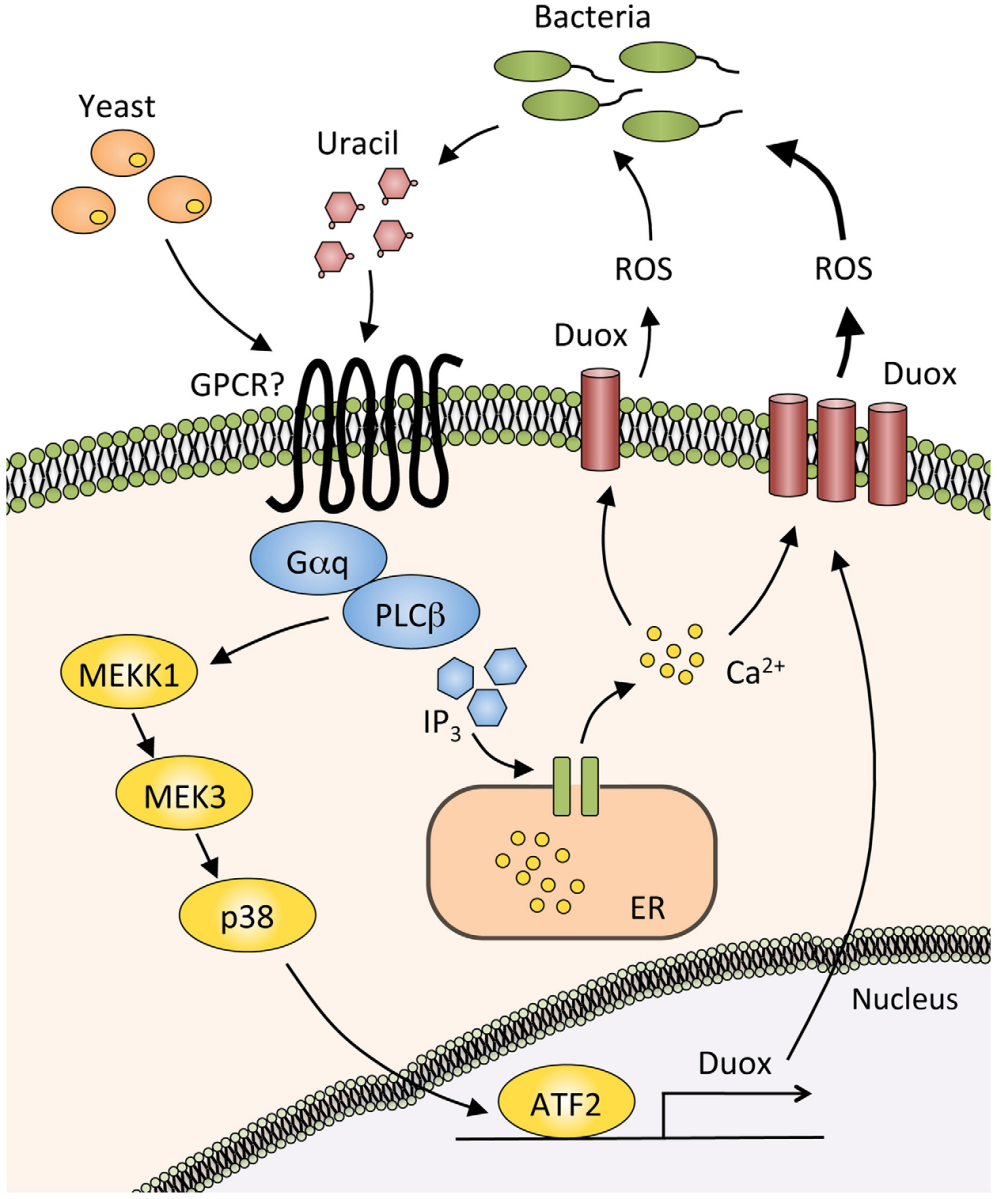
**Production of reactive oxygen species by the Duox pathway.** In basal conditions, dietary yeasts or small amount of uracil from commensal bacteria activate the G protein alpha subunit q (Gαq) and phospholipase Cβ (PLCβ), thereby inducing the synthesis of inositol (1,4,5)-triphosphate (IP_3_), which binds to IP_3_ receptor in the endoplasmic reticulum (ER). Calcium ions are then released from ER into the cytosol, resulting in the activation of a reactive oxygen species (ROS)-producing enzyme Dual oxidase (Duox). Duox expression is strongly induced by the large amount of uracil produced by pathogenic bacteria. PLCβ mediates this Duox upregulation through triggering the MEKK1/MEK3/p38 MAPK pathway and activating Activating Transcription Factor2 (ATF2).

In laboratories, *Drosophila* is reared with yeast-supplemented food, thus the fly is considered to be in a slightly infected condition at all times. Upon routine ingestion of dietary yeasts, phospholipase Cβ (PLCβ) in the gut epithelial cells is activated through the G protein alpha subunit q protein (Gαq), which then mobilizes intracellular Ca^2+^ through inositol (1,4,5)-triphosphate generation. This leads to the enzymatic activation of Duox (Ha et al., 2009a) (Figure 2). Upon severe infection with a heavy microbial burden, the host must produce much more ROS to eliminate pathogens. In this case, Duox expression is increased through triggering the MEKK1/MEK3/p38 pathway, which activates Activating Transcription Factor 2 (ATF2) and eventually induces Duox transcription (Ha et al., 2009b). This MAPK pathway is important for eliminating pathogenic bacteria from the gut.

A recent study revealed that bacterial-derived uracil mediates Duox-dependent ROS production in *Drosophila* gut (Lee et al., 2013). Uracil is released by pathogenic bacteria such as *E. carotovora* and *Gluconobacter morbifer*, and possibly activates an as-yet unidentified G protein-coupled receptor that would be upstream of the Gαq-PLCβ. Symbiotic microbes such as *Lactobacillus plantarum* and *Acetobacter pomorum* have lower ROS-producing effects, probably because those commensals release less uracil. These findings suggest that uracil released by bacteria acts as a factor that classifies microbes as symbiotic or pathogenic.

Although ROS is an important factor for gut homeostasis in *Drosophila*, it damages the gut epithelia upon infection, leading the host to repair this injury by the proliferation and differentiation of intestinal stem cells (ISCs).

## Release of antimicrobial peptides in the gut

AMPs are evolutionarily conserved peptides that can damage and kill microbes, and are found among all classes of life (Zasloff, 2002). The production and regulation of AMPs upon infection are well characterized in *Drosophila*, and AMPs produced by the fat body upon systemic infection in *Drosophila* adults are required for host defense (Lemaitre and Hoffmann, 2007). Two major signaling pathways, the Toll and Imd pathways, control the expression of AMPs in the systemic immune response (Lemaitre and Hoffmann, 2007; Kurata, 2010).

**Figure 3:**
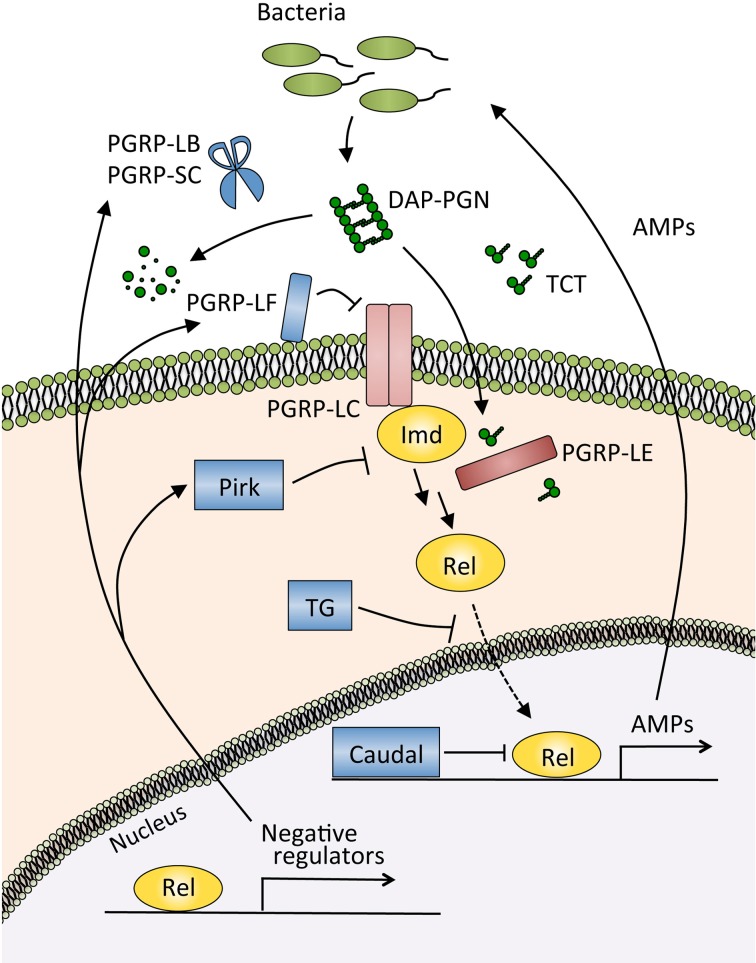
**The Imd pathway and its negative regulators in the gut.** The Imd pathway is activated by the recognition of diaminopimelic acid-type peptidoglycan (DAP-PGN) by the membrane receptor PGRP-LC, or tracheal cytotoxin (TCT) by the intracellular receptor PGRP-LE. Activated PGRP-LC or PGRP-LE then recruits the adaptor protein Imd, which triggers the signaling cascade of the pathway and induces the nuclear translocation of Relish, a nuclear factor-κB (NF-κB) transcription factor of *Drosophila*. The NF-κB protein Relish activates the expression of antimicrobial peptides (AMPs) and negative regulators of this pathway. PGRP-LB and PGRP-SC catalyze the degradation of peptidoglycan and TCT, resulting in a decrease in the amount of ligands for the recognition receptors PGRP-LC and PGRP-LE. PGRP-LF and Pirk inhibit the activity of those receptors, transglutaminase (TG) blocks the nuclear translocation of Relish, and Caudal modulates the transcriptional response of Relish.

In the gut, the Imd pathway controls the generation of AMPs (Tzou et al., 2000; Ryu et al., 2006; Buchon et al., 2009b). This gut immune response is triggered by the recognition of diaminopimelic acid-type peptidoglycan, which is derived from almost all Gram-negative bacteria and a subclass of Gram-positive bacteria, by pattern recognition receptor peptidoglycan recognition protein (PGRP)-LE and PGRP-LC (Bosco-Drayon et al., 2012; Neyen et al., 2012) (Figure 3). Microarray analyses upon the ingestion of *E. carotovora* or *P. entomophila* showed that AMPs and other immune-related molecules are induced in an Imd pathway-dependent manner (Buchon et al., 2009b; Chakrabarti et al., 2012). Flies deficient for Imd pathway activation are more sensitive to oral infection with pathogenic bacteria such as *P. entomophila* and *S. marcescens*, confirming the important contribution of the Imd pathway to host defense in the gut (Liehl et al., 2006; Nehme et al., 2007).

On the other hand, pathogenic bacteria resist the action of AMPs. A genetic study with *P. entomophila* revealed that the *aprA* gene is necessary for the virulence of *P. entomophila*, and a subsequent study identified that *aprA* encodes a secreted metalloprotease that is likely to protect the pathogen against AMPs by deactivating them (Liehl et al., 2006). This study revealed a strategy of pathogenic bacteria to escape from host gut immune responses.

## Gut repair after damage by oral infection

The gut of *Drosophila* is a compartmentalized organ similar to the mammalian intestinal tract (Figure 1A): i.e., its main function is the digestion and absorption of foods, it has a tubular epithelium surrounded by muscles, its activity is partly under the control of the nervous system, and its constant and rapid turnover throughout the lifespan maintain host homeostasis (Cognigni et al., 2011; Buchon et al., 2013b; Marianes and Spradling, 2013). The adult *Drosophila* gut possesses ISCs (Micchelli and Perrimon, 2006; Ohlstein and Spradling, 2006). Their proliferation and differentiation under healthy conditions are regulated by the molecular mechanisms involving the Delta-Notch, Wingless, and Pvr signaling pathways (Micchelli and Perrimon, 2006; Ohlstein and Spradling, 2006; Lin et al., 2008; Bond and Foley, 2012), similar to the mammalian intestine, to maintain its homeostasis. *Drosophila* adult ISCs self-renew and produce two main cell types of the gut: enterocytes that absorb nutrients and enteroendocrine cells that secrete enteric hormones.

**Figure 4:**
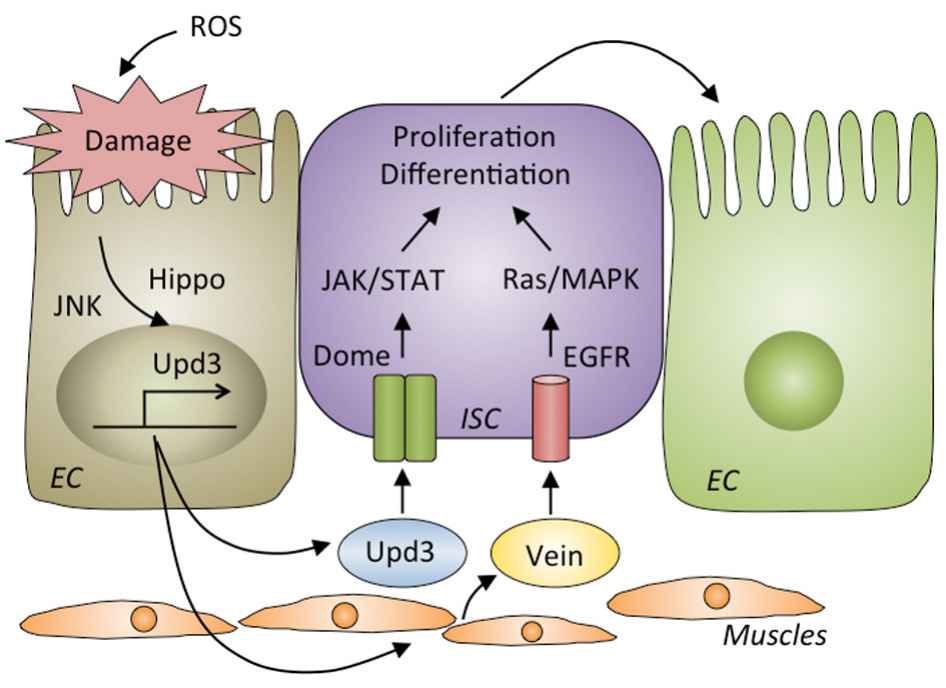
**Gut repair after damage by infection.** Reactive oxygen species (ROS) produced by host cells destroy the gut epithelia. The JNK and Hippo pathways are activated in damaged enterocytes, where they produce Upd3, a ligand for Domeless (Dome) that activates the JAK/STAT pathway in intestinal stem cells (ISCs) to proliferate and differentiate into enterocytes (ECs). Upd3 also triggers the production of Vein in muscle cells that activates EGFR/Ras/MAPK pathway in ISCs to promote their proliferation and differentiation.

Upon oral infection by bacteria, the gut epithelia are damaged and killed by ROS produced by Duox (Buchon et al., 2009a). In fact, oral infection with *E. carotovora* causes the loss of approximately half of the intestinal cells compared to its healthy condition, and a significant shortening of the gut is observed after infection (Buchon et al., 2010). To repair the damage induced by oral infection and maintain gut homeostasis, ISCs are activated to proliferate and differentiate into new enterocytes. The JAK-STAT, epidermal growth factor receptor (EGFR), Hippo and Wingless pathways in the ISCs are required for ISC proliferation and differentiation (Apidianakis et al., 2009; Buchon et al., 2009a,b, 2010; Chatterjee and Ip, 2009; Cronin et al., 2009; Jiang et al., 2009, 2011; Cordero et al., 2012; Zhou et al., 2013) (Figure 4). The JNK and Hippo pathways are activated in damaged enterocytes upon infection (Jiang et al., 2009; Karpowicz et al., 2010; Shaw et al., 2010; Staley and Irvine, 2010; Bond and Foley, 2012). Those enterocytes produce a secreted cytokine, Upd3, a ligand for the receptor Domeless, which activates the JAK-STAT pathway in ISCs to promote both their division and differentiation into enterocytes. Upd3 also acts in visceral muscles to direct the production of an epidermal growth factor, Vein, which triggers the EGFR/Ras/MAPK pathway in ISCs to promote their proliferation. Vein also acts in enterocytes to properly coordinate the exclusion of damaged cells (Buchon et al., 2010; Biteau and Jasper, 2011; Jiang et al., 2011; Zhou et al., 2013). The JAK-STAT and EGFR pathways in ISCs and the EGFR pathway in enterocytes are indispensable for maintaining homeostasis, as flies lacking those pathways in the corresponding cells are highly susceptible to infection (Buchon et al., 2009a, 2010; Jiang et al., 2009; Osman et al., 2012).

Severe infection with the lethal pathogen *P. entomophila* blocks these gut repair pathways. Excessive ROS and pore-forming toxins from *P. entomophila* modulate stress pathways, specifically activation of the GCN2 kinase and inhibition of the target of rapamycin (TOR) pathway, leading to a global translational blockage (Chakrabarti et al., 2012). Consequently, repair cytokines such as Upd3 and Vein are not secreted upon severe infection, leading to failed epithelium renewal and resulting in the death of the fly.

## Commensal flora of the *drosophila* gut

The *Drosophila* gut, like the mammalian intestinal tract, is associated with a number of microorganisms, though the bacterial diversity of flies (~30 different species) is lower than that of mammals (>500 species) (Broderick and Lemaitre, 2012). The most commonly associated bacterial species in flies are members of the *Lactobacillus* and *Acetobacter* groups (Wong et al., 2011). These bacteria have an important role in host physiology, specifically larval growth (Ridley et al., 2012).

Germ-free larvae reared in conditions of nutrient scarcity exhibit delayed development (Storelli et al., 2011). This effect is mediated by *L. plantarum*, as re-introduction of this bacterium into germ-free larvae is sufficient to restore the natural growth rate. *L. plantarum* impacts the host TOR pathway, a major sensor of the nutritional status of the cell, and increases the release of insulin-like peptides into the larval hemolymph.

*A. pomorum* is a member of the gut microbiota that affects larval size and development time under conditions of protein-poor diets (Shin et al., 2011). Bacterial genetic studies revealed that the pyrroloquinoline quinone-dependent alcohol dehydrogenase (PQQ-ADH)-dependent oxidative respiratory chain of *A. pomorum* mediates this effect. Indeed, re-introduction of the PQQ-ADH-deficient *A. pomorum* into the larvae and supplementing the food with acetic acid, a metabolic product of the action of PQQ-ADH, rescues the growth rate of the germ-free larvae.

Gut microbiota likely affect *Drosophila* gut immune responses. *Drosophila* larvae devoid of commensal bacteria are more susceptible than wild-type larvae to infection with *Candida albicans*, suggesting a role of the commensals in host defense (Glittenberg et al., 2011). The molecular mechanism underlying this effect, however, is unclear.

## Negative regulators of the Imd pathway

As discussed above, by regulating ROS production, bacterial-derived uracil is a factor that could explain how flies maintain benign and beneficial bacteria in the gut. In addition, so-called “negative regulators” of the Imd pathway ensure an appropriate level of immune responses in the gut to establish immune tolerance against commensal microorganisms (Lee and Ferrandon, 2011; Kleino and Silverman, 2013). Some of these negative regulators are upregulated by activation of the Imd pathway, establishing a negative feedback loop that adjusts the magnitude of AMP production in the gut.

PGRPs are evolutionally conserved proteins involved in the recognition and degradation of peptidoglycans, a cell wall component of bacteria (Kurata, 2004). There are 13 PGRP family members in the *Drosophila* genome, and 4 family members in humans. PGRP-LB and PGRP-SC, both of which belong to the amidase PGRP family that catalyzes the degradation of peptidoglycan, reduce the levels of Imd pathway-activating ligands in the gut (Bischoff et al., 2006; Zaidman-Rémy et al., 2006; Paredes et al., 2011). Other types of negative regulators directly suppress the activity of the PGRP receptors in the Imd pathway (Figure 3). Membrane-associated protein PGRP-LF probably blocks the dimerization of PGRP-LC, and an intracellular protein Pirk interferes with the interaction of PGRP-LC or PGRP-LE with Imd, thus limiting the activation of the Imd pathway (Aggarwal et al., 2008; Kleino et al., 2008; Lhocine et al., 2008; Maillet et al., 2008; Basbous et al., 2011). The nuclear factor-κB (NF-κB) protein Relish is also controlled by negative regulators. The homeobox protein Caudal, expressed in the posterior midgut, downregulates the expression of AMPs (Ryu et al., 2008). Transglutaminase catalyzes the protein-protein cross-linking of Relish, and the cross-linked Relish has diminished ability to translocate to the nucleus (Shibata et al., 2013).

Flies lacking these negative regulators of the Imd pathway have a shorter lifespan, probably because of the continuous activation of the Imd pathway by the gut microbiota (Paredes et al., 2011). Interestingly, wild-type flies that ingest gut lysates prepared from *transglutaminase*-RNAi flies also have a shorter lifespan (Shibata et al., 2013), suggesting that maintaining the proper gut commensals is crucial to fly survival and that the Imd pathway has an important role in shaping healthy gut commensals.

## Perspectives

Recent studies of the gut immune responses in *Drosophila* revealed resistance and tolerance mechanisms (Ayres and Schneider, 2012) against pathogens: ROS and AMPs kill invading bacteria, the peritrophic matrix diminishes the action of bacterial toxins, and epithelial renewal ensures gut homeostasis after infection-induced damage. To date, most studies have been performed by using Gram-negative bacteria as the pathogens, and thus the mechanisms of coping with oral infection by Gram-positive bacteria, fungi, viruses, and protozoa remain to be clarified. With regard to systemic immune responses, the Toll pathway is required for the defense against Gram-positive bacteria (Lemaitre and Hoffmann, 2007). On the other hand, because of the acidic conditions of the gut and the presence of digestive enzymes, the proteolytic cascades that produce the Toll ligand Spätzle are likely to be inactive in the gut. Thus, Toll signaling does not seem to have a role in gut immunity (Buchon et al., 2009b), suggesting that other effector mechanisms are involved in the gut immune response against Gram-positive bacteria and fungi. Analogously, as a systemic immune response, hemocytes are involved in an encapsulation reaction against parasites intruding into the hemolymph (Lemaitre and Hoffmann, 2007). The observation that hemocytes are likely to be absent in the adult gut lumen implies the existence of distinct defense mechanisms against protozoa in the gut immunity. Gram-positive bacteria *Enterococcus faecalis* and *Enterococcus faecium* cause a gastrointestinal infectious disease in the silkworm. Malaria parasites and dengue viruses invade mosquitos through their gut epithelium (Cirimotich et al., 2010; Lambrechts et al., 2010; Yassine and Osta, 2010). Therefore, deciphering the immune responses to those pathogens in *Drosophila* would be useful for application to other insects of economic or global health importance.

Research of *Drosophila* gut immunity is likely relevant not only to other insects but also to mammals. Recent reports revealed that PGRPs and AMPs in mice significantly contribute to controlling gut microbiota and thereby maintaining intestinal homeostasis. There are four PGRPs in the mouse genome, and mice lacking all these PGRPs are more susceptible than wild-type mice to dextran sulfate sodium (DSS)-induced colitis, which comprises a severe loss of epithelial cells and colon ulceration, and seems to result from the proliferation of more inflammatory gut microflora upon exposure to the DSS (Saha et al., 2010). Similarly, AMPs are important for maintaining healthy microbiota in mice. Angiotensin I converting enzyme (peptidyl-dipeptidase A) 2 (ACE2), with its the renin-angiotensin system-independent function, regulates the homeostasis of dietary amino acid tryptophan, which modulates the expression level of AMPs in the intestine. *ACE2* knockout mice exhibit alterations in AMP expression, which probably affects the ecology of gut microbiota, ultimately leading to the susceptibility to severe DSS-induced colitis (Hashimoto et al., 2012).

Studies of several model animals have revealed that intestinal commensal flora affect host physiology and immunity. Although the mechanisms underlying this phenomenon have been partly revealed, e.g., acetic acid from *A. pomorum* modulates the host insulin pathway to support larval development, further analyses are needed to gain a complete mechanistic understanding of the effect of gut microbiota on the host. Furthermore, how hosts distinguish beneficial bacteria from pathogens remains mostly enigmatic. In *Drosophila*, the amount of uracil released from bacteria is the only factor so far identified to be involved in the bacterial discrimination. Notably, both acetic acid and uracil are small organic compounds. This seems to be reasonable: large molecules, such as cell wall components of bacteria, are commonly shared among bacteria phyla, making it difficult for hosts to distinguish the slight differences between bacterial species or to utilize them as a specific signal from microbes. Instead, bacterial metabolites, particularly secondary metabolites, are often restricted to a narrow set of species, suggesting that the hosts recognize those metabolites as a type of “bacterial signature.” Many conventional studies on gut microbiota in association with nutrient intake and metabolism have examined the diversity and functions of small organic molecules. Investigators of innate immunology acknowledge the importance of bacterial “patterns” that are conserved within a class of microbes and sensed by pattern-recognition receptors. Future studies of gut immunology could target the diverse microbial metabolites as potential mediators of immune response and regulation.

## Author contributions

Takayuki Kuraishi, Aki Hori, and Shoichiro Kurata wrote the paper.

### Conflict of interest statement

The authors declare that the research was conducted in the absence of any commercial or financial relationships that could be construed as a potential conflict of interest.
